# THE PREDICTORS OF SEDENTARY BEHAVIOR AMONG OLDER ADULTS WITH POOR MOBILITY

**DOI:** 10.1093/geroni/igae098.4090

**Published:** 2024-12-31

**Authors:** Albert Okrah, Elizabeth Nelson-Twakor, Sadanand Fulzele, Samantha Landwehr, Karrie Zhao, Katherine Hsieh, Deborah Jehu

**Affiliations:** Augusta University, Augusta, Georgia, United States; Augusta University, Augusta, Georgia, United States; Augusta University, Augusta, Georgia, United States; Georgia State University, Atlanta, Georgia, United States; Georgia State University, Atlanta, Georgia, United States; Georgia State University, Atlanta, Georgia, United States; Augusta University, Augusta, Georgia, United States

## Abstract

Older adults with poor mobility are typically more sedentary, which poses increased risk for disability. Identifying the predictors of objectively measured sedentary behavior may affect targeted treatment strategies. This study aimed to identify the predictors of sedentary behavior in older adults with poor mobility. We recruited 18 older adults with self-reported poor mobility (78.3±8.6 years, 44.4% female) from the community and residential care facilities. Participants reported demographic information (years of education, age, sex, fall history) and completed a battery of assessments, including cognition (Rey Auditory Verbal Learning Test), depression (Geriatric Depression Scale), and gait (gait speed). Participants wore an activity monitor on their non-dominant ankle for 7 days (ActiGraph, Pensacola, FL). We used a minimum wear time of ≥4 days, including 2 weekend days in the analysis. We conducted a linear regression with sedentary behavior percentage as the dependent variable, with moderate-to-vigorous physical activity (MVPA), fall history, depression, memory, and gait speed as variables of interest, and we controlled for age, sex, and education,. All variables were correlated at r≤±0.3. The overall model was significant (R2=0.93, p=0.004). The predictors of high sedentary behavior were: greater depression [β=0.59, CI=0.04,1.14], lower MVPA [β=-0.16, CI=-0.2,-0.13], male sex [β=2.71, CI=0.03,5.39], and better memory [β=1.02, 0.29,1.74]. Our results suggest that older adults with poor mobility who are male, more depressed, less physically active, and have better memory are more at risk for being sedentary. Targeted interventions to reduce sedentary behavior and increase exercise, especially those most at-risk for a sedentary lifestyle, may improve health outcomes.

